# Numerical analysis of airflow alteration in central airways following tracheobronchial stent placement

**DOI:** 10.1186/2162-3619-1-23

**Published:** 2012-08-27

**Authors:** Chien-Yi Ho, Hsiu-Mei Liao, Chih-Yen Tu, Chih-Yang Huang, Chuen-Ming Shih, Min-Ying Lydia Su, Jeon-Hor Chen, Tzu-Ching Shih

**Affiliations:** 1 Graduate Institute of Chinese Medicine, School of Chinese Medicine, China Medical University, Taichung, Taiwan; 2 Department of Family Medicine, China Medical University Hospital, Taichung, Taiwan; 3 Graduate Institute of Basic Medical Science, China Medical University, Taichung, Taiwan; 4 Division of Chest Medicine, Department of Internal Medicine, China Medical University Hospital, Taichung, 40402, Taiwan; 5 Department of Life Science, National Chung Hsing University, Taichung, Taiwan; 6 Department of Respiratory Therapy, China Medical University, Taichung, Taiwan; 7 Preventive Medicine Center, China Medical University Hospital, Taichung, Taiwan; 8 Tu and Yuen Center for Functional Onco-Imaging, Department of Radiological Science, University of California, Irvine, CA, 92697, USA; 9 Department of Radiology, China Medical University Hospital, Taichung, Taiwan; 10 Department of Biomedical Imaging and Radiological Science, College of Health Care, China Medical University, Taichung, 40402, Taiwan

## Abstract

The computational fluid dynamics method, which provides an estimation of the pressure drop in the airway before and after the stent implantation, is proposed in this study. This method is based on the finite volume model. The pressure field was solved by the Navier-Stokes equations. The proposed methodology was evaluated in seven health people (control group) and in fourteen patients who were assigned in two groups, in which one was tracheal stenosis and the other was bronchial stenosis. The results showed that the pressure drop after tracheal stent implantation became significantly smaller. For bronchial stent implantation cases, the airway resistance improved insignificantly.

## Introduction

Airway tumors related to central airway compression produces dyspnea, stridor, hemorrhage, obstructive pneumonia, or combinative syndromes. These patients branded with a poor prognosis may not benefit from surgery with a curative intent; however, they will require procedures for palliation with the hopes of being provided with an improved quality of life. In 1915, Brunings and Albrecht [1] raised the model of endoscopic tracheal stent placement for the therapy of airway strictures. As the development of material technology, the commercial silicon stent which could use rigid bronchoscopy to place the implants was proposed by Duman [2]. From that time, metallic airway stent insertion can achieve symptomatic relief in the majority of patients with airway obstruction, and mounting researches aimed at evaluating the effectiveness of metallic airway stents implants based on the change in lung function [3-8], clinical symptoms [9-12], comorbidity incidence [13,14], and survival rate [15-22], have been reported. However, it was difficult to examine or evaluate the efficacy in some patients with severe airway obstruction [23,24].

Computer tomography (CT) with two-dimensional (2D) or three-dimensional (3D) reconstruction image study can help to evaluate the improvement of the anatomic structure. However, the real alteration of airflow and the spread of gas particles in central airway before and after airway stents placement could not be accurately accessed before the Computational Fluid Dynamics (CFD) software was applied to mimic the internal luminal 3D airflow alteration [16], and to imitate normal human airway internal alterations [25,26]. Xu and Liu [18,21] reported the change in airway flow and pressure during airway obstruction. Kabilan [22] also used the CFD software to record and analyze the sheep airway flow. In 2005, Chen [23] combined 3D computer tomography and CFD software to investigate the coronary artery disease after stent placement. However, there was no report for the airway assessment after stent placement. Thus, the purpose of this study was to set up and evaluate a novel model which is capable of investigating the real airflow dynamic change in central airway obstruction after airway stent implant via combination of CFD technology and 3D computer tomography to mimic numerical simulation of airflow alteration.

## Materials and methods

### Subjects

Our research was reviewed and approved by the Institutional Review Board (IRB) at our institution. Subjects were all recruited at the China Medical University Hospital in Taiwan who underwent tracheobronchial stents (Ultraflex metallic stents, Boston Scientific, Natick, MA) placement in the airway. Twenty-one participants were classified into three groups and diagnostic data were listed in Table 1. The first group was healthy subjects from regular health examination without airway obstruction. The second group was primary or metastatic lung tumor with tracheal obstruction; and the third group was primary or metastatic lung tumor with bronchial obstruction. The recorded clinical data included each patient’s age, gender, clinical symptoms, and the size and location of the stent placement.

**Table 1:** Characteristics of twenty-one subjects

		**Without stenosis**	**Tracheal stenosis**	**Bronchial stenosis**
Gender	M	6	4	6
	F	1	3	1
Median Age(Range)		50.7 (39-66)	63.9 (36-86)	63.4 (51-72)
Diagnosis	Thyroid cancer		1	
	Esophageal cancer		4	
	Breast cancer		1	1
	Lung cancer		1	6
Stent size	20 mm × 4 cm		1	
	18 mm × 6 cm		2	
	16 mm × 6 cm		2	
	16 mm × 4 cm		2	
	14 mm × 3 cm			5
	12 mm × 4 cm			2

### Computed Tomography (CT) images and follow-up

The computed tomographic images of the studied subjects were acquired by the GE Medical Systems, Light Speed or by the GE Medical Systems, Bright Speed. The matrix size was 512× 512, the field of view (FOV) was 360-370 mm. The corresponding volxel size was around 0.7 mm ×0.7 mm × 1.25 mm. The slices of CT images were from 198 to 325. The follow-up time interval for most of the patients receiving stent implantations was about 6 months. In some patients receiving bronchial stent implantation, however, the follow-up time period could be up to 12 months.

### Tracheobronchial stent placement and image software

**Figure 1:**
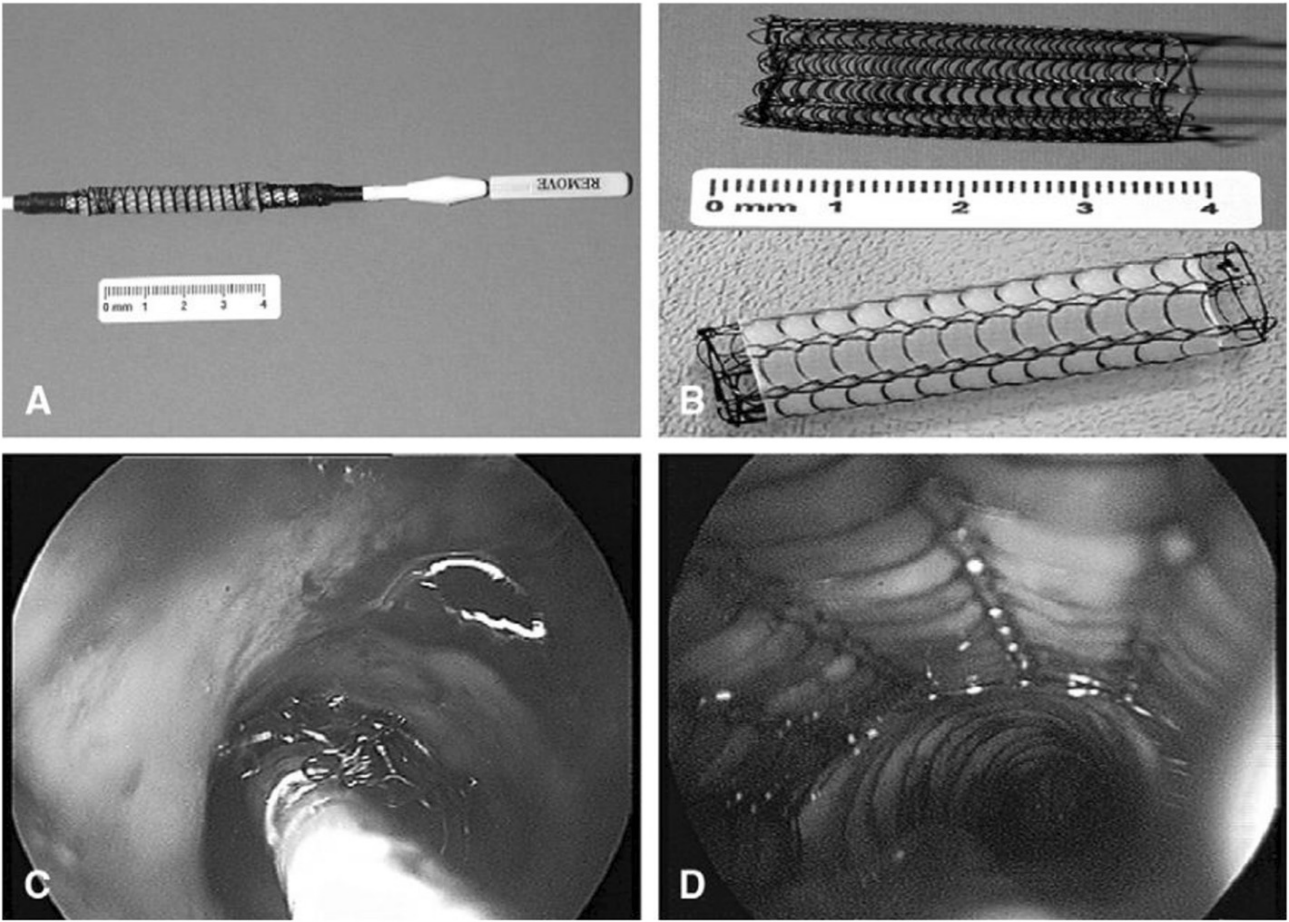
**The implantation process of the Ultraflex stent.** (**A**) Stent bound onto introducer. (**B**) Released standard and covered stents. (**C**) Releasing stent at bronchoscopy. (**D**) Fully released stent.

The tracheal or bronchial stents were placed by flexible bronchoscope (BF-1T260; Olympus; Tokyo, Japan) from mouth to obstruction sites. Prior to the sent placement, each patient received intravenous sedation (midazolam 5 mg) and local anesthetic (2% xylocaine). After evaluation of the chest computed tomography (CT) images, an Ultraflex SEMS (Boston Scientific, Natick, MA, USA) guide wire was inserted from the proximal to distal site of obstruction in the tracheobronchial airway by a chest medicine doctor. The doctor determined the size of the stent and the location of the stent placement by taking into account of the type, length, and location of the airway stenosis. The overall process of stent setting up was shown in Figure 1. In case that the stent must be moved, biopsy pliers (FB-15C-1; Olympus, Tokyo, Japan) were used to grip the end of the stent and to withdraw the bronchoscopy.

### Computational Fluid Dynamics (CFD) modeling

**Figure 2:**
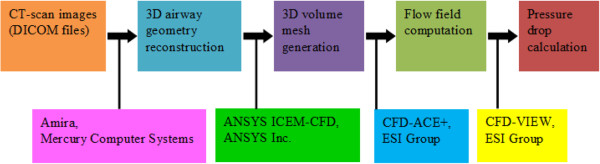
Procedures of numerical simulation.

In order to simulate the airflow in the airway, the CFD technique was used for modeling. In this study, we used the three software packages to reconstruct the 3D airway anatomy geometry, to generate the volume mesh, and to calculate the pressure drop before and after the stent implantation, as shown in Figure 2. Furthermore, the Navier-Stokes equations for the evolution of the airflow in the airway were employed.

First, the segmented images from the original CT (Digital Imaging and Communications in Medicine) DICOM images were processed by the Amira software package. The three-dimensional airway surface reconstruction was created with exclusion of the tracheal cartilage. Second, the volume mesh was generated by the ANSYS CFD ICEM 11.0. Third, the velocity/pressure field in the airway was solved by the CFD-ACE software package.

**Figure 3:**
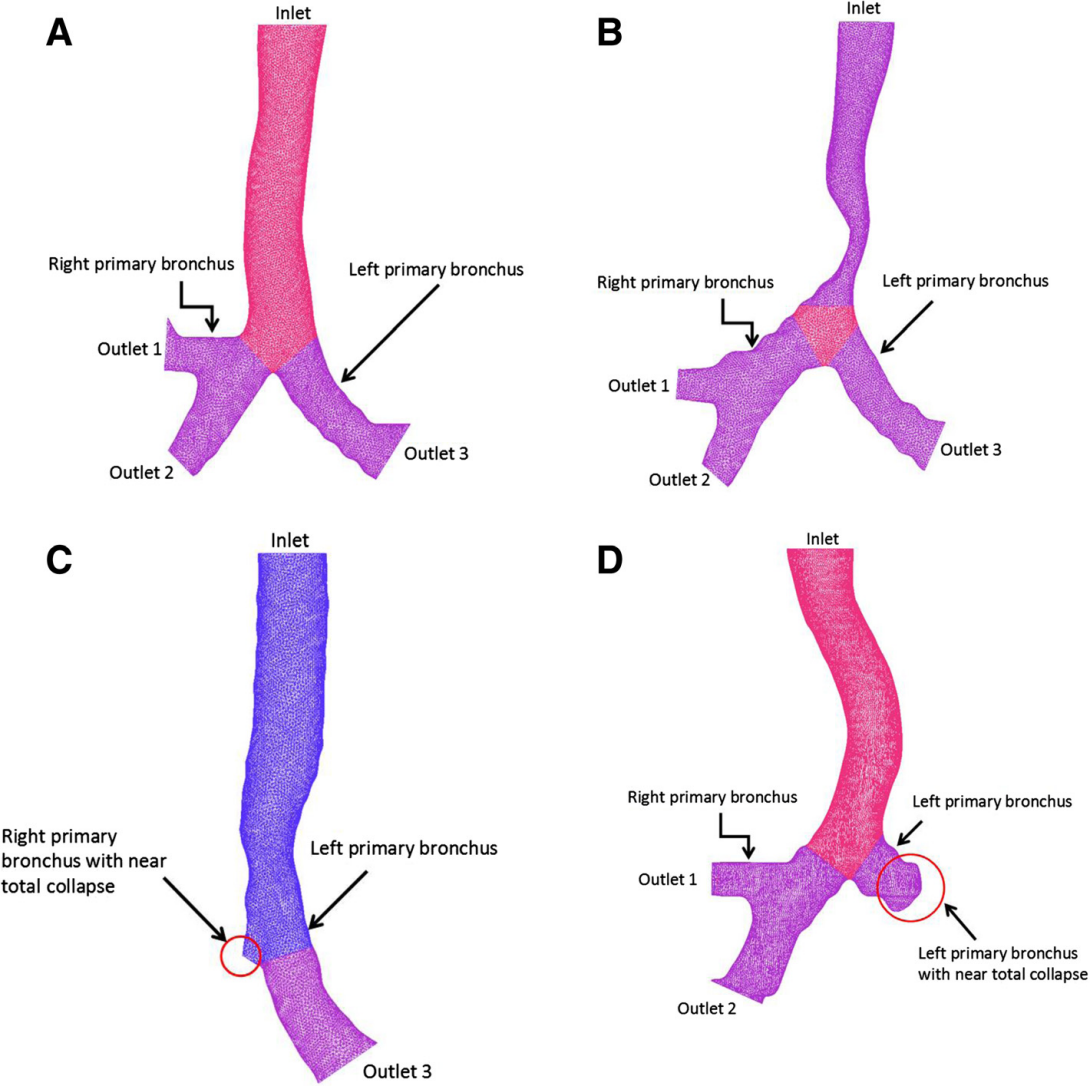
**Boundary conditions were used in numerical simulations.** The constant flow rate of the inlet was set at 100 ml s^−1^. The constant pressures of the three outlets were applied to 0 Pa. (**A**) without stenosis; (**B**) tracheal stenosis; (**C**) bronchial stenosis for the right primary bronchus with nearly total collapse; (**D**) bronchial stenosis for the left primary bronchus with nearly total collapse.

Constant air pressure was imposed at the outlet when the constant flow rate was applied at the inlet. The wall of the airway was assumed as the no-slip boundary (i.e., the speed on the wall surface is zero). The viscosity of air was set to 1.864.10^-5^ kg m^-1^s^-1^ in numerical simulation. According to the airflow rate of van Ertbruggen et al. [17], the airflow rate of the inlet was set at 100 ml s^-1^ and the constant pressure of the outlet was applied to 0 Pa in this study. The boundary conditions of the CFD model were shown in Figure 3. With the length from about 10 cm above the carina, the viscous pressure drop in the 10-cm segment of the airway was used for evaluating the airflow resistance for a respiration process.

## Results

First, we tested the CFD model in 7 healthy subjects (i.e., without airway stenosis). We analyzed the changes of the cross-sectional area of the inlet and outlet and pressure drop in the airway for these 7 subjects (the inlet flow rate was set to 100 ml s^-1^ and the outlet pressure was set to 0 Pa) (Table 2). In these cases, the Reynolds number (Re) was 492 ± 101 (range: 382 ~ 677). The mean pressure drop of the inlet was 0.70 ± 0.41 Pa. The average airflow rates of the right primary and left bronchus were 1.06 ± 0.66 ml s^−1^ and 0.81 ± 0.63 ml s^−1^, respectively. In addition, the ratio of the inflow air to the right lobe and left lobe were 56.57% and 43.43%, respectively.

**Table 2:** Cross-sectional areas and pressure drops for 7 healthy subjects

**Subject**	**Cross-sectional area (cm**^**2**^**)**						**Pressure drop (Pa)**		
	**Inlet**	**Right primary bronchus**	**Left primary bronchus**	**Outlet 1**	**Outlet 2**	**Outlet 3**	**Inlet**	**Right primary bronchus**	**Left primary bronchus**
1	3.06	2.21	1.65	0.99	1.28	1.46	0.59	0.15	0.15
2	2.05	1.68	1.34	0.67	0.90	1.42	0.66	0.29	0.29
3	3.45	1.93	1.65	0.55	1.00	1.04	0.63	0.28	0.29
4	3.14	0.87	1.37	1.88	2.08	2.30	0.34	0.07	0.07
5	1.33	1.57	1.16	0.83	0.51	1.19	1.49	0.51	0.49
6	2.12	2.02	1.35	0.53	0.82	1.02	0.91	0.44	0.47
7	4.17	2.78	2.70	0.99	1.35	2.10	0.26	0.10	0.10
Average	2.76	1.87	1.60	0.92	1.13	1.50	0.70	0.26	0.27

The changes of the cross-sectional area and pressure drop before and after the tracheal stent implantations were listed in Table 3. The average values of the cross-sectional area before and after stenting implantations were 1.45 ± 0.51 cm and 1.47 ± 0.86 cm, respectively. The average pressure drops before and after stenting placement were 10.61 ± 9.22 Pa and 1.71 ± 1.31 Pa. Before the tracheal stent placement, the average airflow percentages of the right primary bronchus and the left primary bronchus were 48.43% and 51.57%. In contrast, after tracheal stenting, the average flow percentages of the right primary and left primary bronchi were 57.71% and 42.29%, respectively. The percentages of 57.71% and 42.29% were similar to that of the healthy cases (56.57%: 43.43%). In other words, the fraction of the airway flow of the right primary bronchus and the left primary bronchus is similar to the healthy human condition after the tracheal stent implantation.

**Table 3:** Cross-sectional areas and pressure drops for 7subjects with tracheal stenosis before and after tracheal stent implantations

**Subject**		**Cross-sectional area (cm**^**2**^**)**						**Pressure drop (Pa)**		
		**Inlet**	**Right primary bronchus**	**Left primary bronchus**	**Outlet 1**	**Outlet 2**	**Outlet 3**	Inlet*	**Right primary bronchus**	**Left primary bronchus**
1	Before	0.90	1.98	2.54	0.42	0.45	1.31	26.66	0.61	0.52
	After	0.53	1.22	0.81	0.53	0.46	1.35	3.96	0.39	0.32
2	Before	1.58	1.61	1.28	1.08	1.64	1.31	4.85	−0.04	−0.01
	After	2.24	1.79	1.48	0.99	1.79	1.71	0.59	0.07	0.07
3	Before	1.69	1.37	0.97	0.33	0.78	0.76	6.46	0.26	0.59
	After	2.66	1.71	1.62	0.42	1.49	1.33	1.44	0.40	0.39
4	Before	1.98	1.01	0.93	0.21	0.44	1.79	4.73	0.11	0.59
	After	1.96	1.00	1.17	0.66	2.04	2.35	0.84	0.14	0.24
5	Before	0.73	1.97	0.90	1.03	0.70	1.45	8.92	0.23	0.02
	After	1.02	1.75	1.12	0.97	1.47	1.38	0.68	0.39	0.45
6	Before	1.25	1.42	1.76	0.86	0.81	1.33	2.25	0.27	0.33
	After	0.39	1.73	1.54	0.95	0.46	1.52	1.65	0.18	0.23
7	Before	2.07	1.40	1.44	0.37	0.62	0.98	20.36	−0.20	0.41
	After	1.53	1.16	1.43	0.43	1.40	1.12	3.15	0.27	0.20
Average	Before	1.45	1.53	1.40	0.61	0.77	1.27	10.60	0.17	0.35
	After	1.47	1.48	1.31	0.70	1.30	1.53	1.75	0.26	0.27

*: The airflow rate of the inlet was set to 100 ml s^-1^.

For 7 patients with bronchial stent placements, the values of the cross-sectional areas of the inlet before and after stenting were 2.81 ± 0.33 cm and 2.63 ± 0.24 cm respectively. There is a small difference of the area between before and after bronchial stenting implantations. The average pressure drop before bronchial stent placement was 0.97 ± 0.55 Pa. After bronchial stent placement, the average pressure drop was 1.24 ± 0.97 Pa after placement. When comparing the 7 healthy subjects and the 7 patients with bronchial stenosis diseases, the pressure drop for the patients before bronchial stent implantation was greater than that of the healthy people 0.70 Pa v.s. 1.24 Pa. On the other hand, the airway resistance was higher in patients with bronchial stenosis even after bronchial stent implementations.

In the clinical measurements, we did pulmonary function test (PFT), including FEV1, PEF (i.e., peak expiratory flow reading, which is lower when the airway is constricted), FEV1%, and FEV1/FVC ratio (i.e., Tiffeneau index, which is used in the diagnosis of obstructive and restrictive lung disease) for some patients with stent placements. The results of two patients correlated with the results of numerical analysis of airflow alteration were given here. For the tracheal stenosis (subject #3 in Table 3), for example, the 80-year-old male patient was diagnosed with the lung cancer of the left upper lobe with the lower third tracheal invasion and stridor symptom and the size of the airway stent was 40 mm by 16 mm. Before the tracheal stent placement, the FEV1/FVC ratio, FEV1, FEV1%, and PEF for subject # 3 were 40.65%, 0.74 L, 37%, and 15.9%, respectively. The FEV1/FVC ratio of the patient was less than 70% and the patient was usually diagnosed as the chronic obstructive pulmonary disease (COPD). The air trapping (i.e., gas trapping, which is an abnormal retention of air in the lungs where it is difficult to exhale completely.) was also observed in this patient. The value of the PEF reading for subject #3 was significantly less than 50%. Thus, the patient has a severe airway narrowing. After the two days of the stent placement, the FEV1/FVC, FEV1, and FEV1% were 69.79%, 1.75 L, and 87.1%, respectively. The stridor symptom of the patient was significantly improved. In CFD simulations, we also found that the pressure drops were 6.46 and 1.44 Pa before and after the stent placement, respectively. In other words, the airway resistance was significantly reduced after the stent placement.

For the bronchus stenosis with the stent placement (subject #2 in Table 4), for instance, the 69-year-old female patient was diagnosed with the breast cancer with the right main bronchus metastasis. The patient was treated by the right primary bronchial stent placement and the stent size was 30 mm by 14 mm. The measurement values of the FEV1/FVC ratio, FEV1, and FEV1% of the patient were 53.5%, 0.92 L, and 59.6%, respectively. After the bronchial stent implantation, the measurement values of the FEV1/FVC ratio, FEV1, FEV1%, and PEF became 74.6%, 1.83 L, 118.2%, and 63%, respectively. The FEV1 value of the patient was changed from 0.92 L to 1.83 L. From CFD simulations, the pressure drops of the right primary bronchus were 0.31 and 0.07 Pa before and after the right bronchial stent placement, respectively. At the meantime, the pressure drop of the left primary bronchus varied from 0.19 Pa to 0.09 Pa after the right primary bronchus. Therefore, the CFD numerical results are consistent with the clinical measurements. The comparison of CFD simulation results and PFTs before and after treatment of the airway stent placement was shown in Table 5 (in the last page of the manuscript).

**Table 4:** Cross-sectional areas and pressure drops for 7subjects with bronchus stenosis before and after bronchial stent implantations

**Subject**		**Cross-sectional area (cm**^**2**^**)**						**Pressure drop (Pa)**		
		Inlet*	**Right primary bronchus**	**Left primary bronchus**	**Outlet 1**	**Outlet 2**	**Outlet 3**	**Inlet**	**Right primary bronchus**	**Left primary bronchus**
1	Before	2.94	2.72	1.48	1.06	1.89	0	0.65	0.28	0.40
	After	2.40	2.44	2.20	0.96	1.92	0.89	0.35	0.17	0.23
2	Before	2.77	0.23	1.62	0.97	1.06	1.82	1.05	0.31	0.19
	After	3.00	0.64	1.31	0.84	0.91	1.73	1.17	0.07	0.09
3	Before	3.38	2.50	2.46	0.79	1.00	1.11	0.50	0.33	0.39
	After	2.89	2.83	1.91	0.98	0.96	0.69	0.50	0.34	0.38
4	Before	2.17	0.75	1.85	0	0	1.59	2.12	2.05	1.82
	After	2.44	0.76	1.67	0.07	0.45	1.66	1.74	1.57	1.45
5	Before	2.80	1.59	2.00	0	0.84	1.60	0.89	0.57	0.47
	After	2.58	1.03	0.99	0	0.45	1.25	3.36	2.44	2.79
6	Before	2.65	1.75	1.59	0.48	0.71	2.02	0.54	0.44	0.30
	After	2.34	1.56	1.27	0.44	0.44	1.77	0.83	0.64	0.41
7	Before	2.95	1.80	1.97	0.42	1.14	0	1.05	0.62	0.82
	After	2.80	1.66	1.27	0.36	1.40	1.21	0.62	0.29	0.38
Average	Before	2.84	1.62	1.85	0.53	0.94	1.16	0.97	0.65	0.62
	After	2.63	1.56	1.51	0.52	0.93	1.31	1.24	0.78	0.81

**Table 5:** Comparison of CFD simulation results and pulmonary function tests before and after treatment of the airway stent placement

	**Diagnosis**	**Sex**	**Age**	**Stent**	**Treatment**	**FEV1 (L)**	**FEV1% (%)**	**FEV1/FVC ratio (%)**	**Pressure drop (Pa)**	**Area (cm**^**2**^**)**
*Tracheal stenosis	Lung cancer	Male	80	40 mm ×16 mm						
					Before	0.74	37	40.65	6.46	1.69
					After	1.75	87.1	69.79	1.44	2.66
**Bronchial stenosis	Breast cancer	Female	69	30 mm ×14 mm						
					Before	0.92	59.6	53.5	1.05	2.77
					After	1.83	118.2	74.6	1.17	3.00

*Tracheal stenosis: subject #3 in Table 3.

**Bronchial stenosis: subject #2 in Table 4.

## Discussion

This study used computational fluid dynamics method, based on the finite volume model, to study the pressure change of the tracheobronchial tree before and after the stent placement. After tracheal stent implantations, the ratio between the right lung and the left was 57.71% to 42.29%, which was similar in healthy people. The ratio of airflow in the right lung and the left lung is 56:44 in normal people [17,27]. We found that the tracheal stent placement can enlarge the lumen of the trachea to improve airway symptom. The pressure drop was significantly decreased after the stenting implantation. Smaller pressure drop has a smaller airway resistance. Vergnon [3] found that the peak expiratory flow rate was decreased when the airflow resistance was increased in patients with central airway obstruction. In our study, all the clinical respiratory distress symptoms of the patients treated by airway stent implantation had been improved.

Furthermore, we also found that the airflow in the airway was significantly improved after the tracheal stent implantation for the patient with the tumor-related central airway obstruction. In those patients with airway tumors that compressed the tracheas, the luminal cross-sectional area of the trachea became smaller and the airway resistance was increased. Although the airway stent placement cannot significantly enlarge the narrowed luminal area in some cases, it effectively decreases the airway resistance. The airflow pattern returns nearly to that of healthy people. For the tracheal stent implantation cases, the average flow of the right and left main bronchus are 57.71% and 42.29%, respectively. In other words, the airflow ratio of the right lung and the left lung of the patients with the tracheal stent implantations was similar to those of the healthy people without tracheal stenosis (i.e., 56.58% and 43.42%).

For the bronchial stent implantation cases, the pressure drop of the airway changed insignificantly. Miyazawa [28] also demonstrated the same situation because the bronchus still collapsed in obstructive lung disease even with the bronchial stenting. In addition, in these cases the turbulent airflow decreased, but did not reach the normal respiratory conditions.

In this study all the 14 patients were originally arranged to have the pulmonary function tests but not all of them can be completed for the study due to the general poor condition and dyspnea of the patients. Eventually only eight patients had pre-stent PFTs, five had post-stent PFTs, and four had both pre- and post-stent PFTs for comparison. From the two examples shown in Table 5, it was noted that the CFD numerical results are consistent with the clinical measurements.

There are some limitations of this pilot study. First, the case number is small and larger numbers are urgently needed for further evaluation. Second, the limited cases are of different tumor types and cancer stages so that the temporarily analyzing classification may not be so conclusive. Third, the lack uniform following up time schedule in this retrospective study, such as the time of computed tomography, may also cause some errors in the analysis.

## Conclusion

In conclusion, we provide a comprehensive model to systematically evaluate patients with obstructive lung tumor. From the limited examples we have found that CFD numerical results correlated well with the results of PFTs, indicating that CFD can potentially become a clinical prognosis prediction tool to assess lung cancer patients who have poor general condition and PFTs cannot be successfully applied to. Also, we found that tracheobronchial stent placement indeed improve cancer patients’ quality of life by lowering the resistance in their respiratory tract. The model is very helpful in not only evaluating the outcome of personalized stent placement but following up of each patient undergoes tracheal and/or bronchial stent placement.

## Competing interests

All the authors declare that they have no competing interests.

## Authors’ contributions

CYH designed the case-control study used for this analysis, literature review, interpreted the results, and drafted the manuscript. HML and TCS ran the computer algorithm and did the computational fluid dynamics simulation of the airflow field in the airway. CYT and CMS recruited subjects and performed airway stent placement studies. CYH, MYLS and JHC designed the case-control study and interpreted the results. All authors read and edited the manuscript, and approved the final manuscript submitted for publication.
